# α-Synuclein disrupts the anti-inflammatory role of Drd2 via interfering β-arrestin2-TAB1 interaction in astrocytes

**DOI:** 10.1186/s12974-018-1302-6

**Published:** 2018-09-10

**Authors:** Ren-Hong Du, Yan Zhou, Mei-Ling Xia, Ming Lu, Jian-Hua Ding, Gang Hu

**Affiliations:** 1 Department of Pharmacology, Jiangsu Key Laboratory of Neurodegeneration, Nanjing Medical University, 101 Nongmian Avenue, Nanjing, Jiangsu 211166 People’s Republic of China; 2 Department of Pharmacology, Nanjing University of Chinese Medicine, 138 Xianlin Avenue, Nanjing, Jiangsu 210023 People’s Republic of China; 3 Department of Clinical Pharmacy, Shanghai General Hospital, Shanghai Jiao Tong University School of Medicine, Shanghai, 200080 People’s Republic of China

**Keywords:** α-Synuclein, Dopamine D2 receptor, β-Arrestin2, Inflammation, Astrocyte, Parkinson’s disease

## Abstract

**Background:**

α-Synuclein (α-Syn)-induced neuroinflammation plays a crucial role in the pathogenesis of Parkinson’s disease (PD). Dopamine D2 receptor (Drd2) has been regarded as a potential anti-inflammatory target in the therapy of neurodegenerative diseases. However, the effect of astrocytic Drd2 in α-Syn-induced neuroinflammation remains unclear.

**Methods:**

The effect of Drd2 on neuroinflammation was examined in mouse primary astrocyte in vitro and A53T transgenic mice in vivo. The inflammatory responses of astrocyte were detected using immunofluorescence, ELISA, and qRT-PCR. The details of molecular mechanism were assessed using Western blotting and protein-protein interaction assays.

**Results:**

We showed that the selective Drd2 agonist quinpirole suppressed inflammation in the midbrain of wild-type mice, but not in α-Syn-overexpressed mice. We also found that Drd2 agonists significantly alleviated LPS-induced inflammatory response in astrocytes, but failed to suppress α-Syn-induced inflammatory response. The anti-inflammation effect of Drd2 was dependent on β-arrestin2-mediated signaling, but not classical G protein pathway. α-Syn reduced the expression of β-arrestin2 in astrocytes. Increased the β-arrestin2 expression restored in the anti-inflammation of Drd2 in α-Syn-induced inflammation. Furthermore, we demonstrated that α-Syn disrupted the anti-inflammation of Drd2 via inhibiting the association of β-arrestin2 with transforming growth factor-beta-activated kinase 1 (TAK1)-binding protein 1 (TAB1) and promoting TAK1-TAB1 interaction in astrocytes.

**Conclusions:**

Our study illustrates that astrocytic Drd2 inhibits neuroinflammation through a β-arrestin2-dependent mechanism and provides a new strategy for treatment of PD. Our findings also reveal that α-Syn disrupts the function of β-arrestin2 and inflammatory pathways in the pathogenesis of PD.

## Background

Parkinson’s disease (PD), the second most common neurodegenerative disorder after Alzheimer’s disease, is characterized by the progressive loss of dopaminergic (DA) neurons in substantia nigra compacta (SNc), accumulation of α-synuclein (α-Syn) in Lewy bodies and neurites, and excessive neuroinflammation [1]. Neuroinflammation is mediated predominantly by activated glial cells and is accompanied by the production of inflammatory cytokines [2]. Microglia has long been considered a key player in neuroinflammation [3, 4]. Increasing evidence suggests that activated astrocytes play a vital role in neuroinflammation in the aging brain and most neurodegenerative diseases (NDDs) including PD [5–7]. Activated astrocytes release pro-inflammatory cytokines and chemokines, which may induce neuronal damage. Although the exact pathogenic mechanisms of PD remain unclear [8], reactive astrogliosis and astrocyte-mediated neuroinflammation have been recognized to play critical role in the pathogenesis of PD [9, 10].

α-Syn is the principal component of Lewy pathology and strongly influences on the pathogenesis of PD [11]. Although α-Syn deposits are primarily found in neurons in the PD brain, they also appear frequently in astrocytes [12, 13]. Besides directly damaging mitochondria and lysosome of neurons, the increased level of α-Syn also causes glial response and the reactive glia release inflammatory mediators such as reactive oxygen species, nitric oxide, tumor necrosis factor-α (TNF-α), and interleukin-1β (IL-1β) [14, 15]. They are the main causes of synaptic dysfunction and neuronal death, leading to a self-amplifying cycle of inflammation, and eventually accelerate the process of PD [16]. Besides reactive microglia along with Lewy bodies that found in the substantia nigra of PD patients, it is now increasingly appreciated that astrocytes also function in initiating and perpetuating inflammatory process associated with PD [17, 18]. Thus, controlling astroglial-mediated neuroinflammation may prove to be a promising therapeutic strategy for PD.

Dopamine D2 receptor (Drd2) has been regarded as a potential anti-inflammatory target in the therapy of NDDs [19–21]. Drd2 expresses both in neurons and astrocytes [22]. Our previous study revealed that astrocytic Drd2 controls the expression of anti-inflammatory protein αB-crystallin and helps maintaining the immune homeostasis in the CNS [23]. However, the relation of astrocytic Drd2 and α-Syn-induced neuroinflammation has not been defined yet. Drd2 couples with Gαi protein and participates to trigger two types of signal transduction pathways: the classic G protein-dependent pathway and the β-arrestin-dependent pathway [24, 25]. β-arrestin2 is a critical adaptor and regulator of internalization and desensitization of G protein-coupled receptors (GPCR) [25]. It can also serve as a signal effector itself, regulating inflammatory response [26]. It was reported that β-arrestin2 was necessary to mediate the anti-inflammation function of β2 adrenergic receptor and kappa-opioid receptor in microglia [27, 28]. However, its role in astrocytes remains unknown and the precise mechanism by which β-arrestin2 mediates GPCR-related anti-inflammation role is still poorly understood.

In the present study, we investigate the effect of astrocytic Drd2 on α-Syn-induced neuroinflammation in astrocytes and in A53T transgenic (A53T^tg/tg^) mice. Surprisingly, here we showed that Drd2 activation significantly inhibited lipopolysaccharide (LPS)-induced inflammation, but failed to suppress α-Syn-induced inflammatory response in vivo and in vitro. We further demonstrate that β-arrestin2 is critical for anti-inflammation of Drd2 in astrocytes. α-Syn abolishes the anti-inflammation role of Drd2 via suppression of the β-arrestin2-transforming growth factor-beta-activated kinase 1-binding protein 1 (TAB1) interaction in astrocytes.

## Methods

### Mice

A53T transgenic (A53T^tg/tg^) mice [29, 30] on a C57BL/6 background were obtained from Model Animal Research Center of Nanjing University. Genotypes were confirmed by real-time PCR. A53T^tg/tg^ mice and their littermate wild-type (WT) controls were bred and maintained in the Animal Resource Center of the Faculty of Medicine, Nanjing Medical University and age-matched adult male mice (4 month old) used for the experiments. All mice were maintained under specific pathogen-free conditions and were treated in accordance with protocols approved by the Institutional Animal Care and Use Committee of Nanjing Medical University.

### Primary astrocyte culture and treatment

Primary astrocyte was prepared from the midbrain of WT and A53T^tg/tg^ mice at P0–3, as described previously [31]. The neonatal midbrain were trypsinized and dissociated, and cells were plated at density of 2 × 10^6^ cells in DMEM/Ham’s F12 medium containing 10% FBS on 6-well culture plates. Culture media were changed 24 h later to complete medium and subsequently twice a week. The purity of astrocyte was > 95% as determined with GFAP immunocytochemistry. Before experimental treatments, astrocytic cultures were passaged once. Astrocytes were pretreated with quinpirole (10 μM, TOCRIS) for 1 h before LPS (100 ng ml^−1^, Sigma-Aldrich) or α-Syn (10 μg ml^−1^, Sigma-Aldrich) stimulation for 24 h.

### Mesencephalic primary neuron cultures and treatment

Mesencephalic primary neuron cultures were prepared from the ventral mesencephalic tissues of C57BL/6 mice on embryonic day 14/15 (E14/15), as described previously [32]. LPS or α-Syn-stimulated astrocytic conditioned medium was collected and centrifuged at 1000 g for 5 min to remove debris and dead cells. Mesencephalic primary neurons were incubated with the supernatant mixed with neurobasal medium at a ratio of 1:2 for 6 h before immunocytochemical staining.

### Immunocytochemical staining

After incubation with conditioned medium generation from astrocytes for 6 h, primary dopaminergic neurons were identified by immunocytochemical staining of tyrosine hydroxylase (TH). Cells were rinsed with 0.1 M PBS carefully and fixed with 4% paraformaldehyde, followed by incubation with 3% H_2_O_2_ to remove the endogenous peroxidase. After washing, cells were blocked with PBS containing 5% bovine serum albumin (BSA) and then incubated with the primary antibody (T2928, the mouse monoclonal anti-TH at 1: 4000; Sigma) at 4 °C overnight. After washing, cells were exposed to HRP-conjugated second antibody (goat anti-mouse at 1:800; KPL) for 1 h at room temperature. Immunostaining was visualized using DAB-H_2_O_2_ (Boster).

### Quantification of TH-positive cell count and neuronal processes

TH-positive neurons were counted in 10 randomly selected fields at × 100 magnification by the Nikon Optical TE2000-S inverted microscope. The number of TH-positive cells accounted for about 3% of total cells in the primary cultures. Cell processes were measured at × 200 magnification. Thirty TH-positive cells were selected at random for each treatment. TH-positive cell processes were traced from cell body to the end, and the total length was quantitated using Image Pro Plus 5.1. Only processes that more than twice the length of the cell body were measured.

### In vivo experimental treatments

A53T^tg/tg^ and WT mice were administered intraperitoneal injections of 5 mg kg^−1^ quinpirole per day for 10 day. WT mice were given intraperitoneal injections of l-methyl-4-phenyl-l, 2, 3, 6-tetrahydropypridine (MPTP; 30 mg kg^−1^) for four consecutive days and left for 3 days. At day 7 post-injection, the animals were killed and the SNc were dissected and processed for Western blot or Elisa analysis.

### Western blotting analysis

Cell lysates and tissues were homogenized in lyses buffer (Beyotime, China), and protein concentration was determined by the Bradford assay (Bio-Rad, Hercules, CA). The analysis of protein was performed according to standard SDS-PAGE. After blocking, the membranes were incubated with various specific primary antibodies against β-arrestin2 (3857, 1:800), TLR4 (14358, 1:1000), phospho-IKKβ (2697, 1:1000), IKKβ (8943, 1:1000), phospho-TAK1 (4508, 1:1000), TAK1 (5206, 1:1000), TAB1 (3225, 1:1000), H3 (4499, 1:1000), phospho–p65 (3033, 1:1000), p65 (8242, 1:1000), β-actin (3700, 1:5000) from Cell Signaling Technology, D2R (AB1558, Millipore, 1:1000), and α-synuclein (610786, 1:1000, BD Biosciences) in TBST at 4 °C overnight. After washing, the bands were incubated with corresponding horseradish peroxidase–conjugated secondary antibodies (Santa Cruz Biotechnology, USA) for 1 h at room temperature, and signals were detected by enhanced chemiluminescence (ECL) Western blot detection reagents (Pierce, Rockford, IL). The membranes were scanned and analyzed using ImageQuant™ LAS 4000 imaging system (GE Healthcare, Piscataway, NJ, USA).

### ELISA

Cell culture supernatants and SNc were assayed for IL-1β (Mouse IL-1 beta/IL-1F2 Quantikine ELISA Kit, MLB00C**)**, TNF-α (Mouse TNF-alpha Quantikine ELISA Kit, MTA00B), IL-6 (Mouse IL-6 Quantikine ELISA Kit, M6000B), and IL-12 (Mouse IL-12 p70 Quantikine ELISA Kit, M1270) with ELISA kits from R&D Systems according to the manufacturer’s instructions.

### Reverse transcription and quantitative real-time PCR

Total RNA was extracted from astrocytes using TRIzol Reagent (Invitrogen Life Technologies), and RNA concentrations were determined by ultraviolet spectrophotometry (Nano VueTM). Reverse transcription was performed with PrimeScript™ 1st Strand cDNA Synthesis Kit (Takara) according to standard protocol. Quantitative real-time PCR was performed on an ABI 7300 Real-Time PCR System (Applied Biosystems, Japan) using the Fast Start Universal SYBR Green Master (Rox) (Roche Diagnostics Ltd.). GAPDH was used as an endogenous control. Polymerase chain reaction (PCR) primers were used as follows: GAPDH sense: 5′-CAAAAGGGTCATCATCTCC-3′, antisense: 5′-CCCCAGCATCAAAGGTG-3′, IL-1β sense: 5′-TCATTGTGGCTGTGGAGAAG-3′, antisense: 5′-AGGCCACAGGTATTTTGTCG-3′, TNF-α sense: 5′-CATCTTCTCAAAATTCGAGTGACAA-3′, antisense: 5′-TGGGAGTAGACAAGGTACAACCC-3′, IL-6 sense: 5′-ATCCAGTTGCCTTCTTGGGACTGA-3′, antisense: 5′-TAAGCCTCCGACTTGTGAAGTGGT-3′, IL-12 sense: 5′-GCCAGGTGTCTTAGCCAGTC-3′, antisense: 5′-CAGATAGCCCATCACCCTGT-3′, IFN-γ sense: 5′-TGGCATAGATGTGGAAGAAAAGAG-3′, antisense: 5′-TGCAGGATTTTCATGTCACCAT-3′, D_2_R sense: 5′-ATCTCTTGCCCACTGCTCTTTGGA-3′, antisense: 5’-ATAGACCAGCAGGGTGACGATGAA-3′.

### Co-immunoprecipitation (Co-IP)

The total cell lysate prepared from astrocytes was incubated with anti-TAB1 antibody followed by incubated with protein A/G plus agarose (Santa Cruz Biotechnology) as described previously [33]. After washing the beads, the bound proteins were eluted and analyzed by immunoblotting.

### Radioligand binding assays

WT and A53T^tg/tg^ mice of 4-month-olds were sacrificed and synaptosomes (homogenization of fresh brain tissue in isotonic medium shears plasma membranes causing nerve terminals to become separated from their axons and postsynaptic connections. The nerve terminal membranes then reseal to form synaptosomes. The purified synaptosomes are viable and take up and release neurotransmitters very efficiently) of the striatum and were rapidly separated on ice. Wet tissues were weighed and homogenized in 5 × weight/volume (*w*/*v*) icy sucrose buffer (0.32 M sucrose, 0.01 M HEPES, pH 7.4) followed by centrifugation at 1000 g for 10 min. The supernatant was further centrifugated at 17000 g for 20 min, and the pallet was resuspended in Aaasy Buffer (50 mM Tris, 5 mM KCl, 2 mM NaCl, 2 mM CaCl_2_, 1 mM K_2_PO_4_, and pH 7.3, 4 °C). Samples of the cell fractions (100 μl of each) were assessed for protein content by Biuretassay, and the remainder stored at − 80 °C for use in subsequent radioligand binding studies.

[^3^H] Spiperone (Perkin Elmer, 1187001MC) was used as the radioligand in the radioligand binding assay. Samples were incubated with a range of radioligand concentrations (0~1 nM) at 37 °C. The specific binding component was determined using (+)-Butaclamol (1 μM/5 μM). Assays were terminated by ice-cold wash buffer (150 mM Tris HCl, pH 7.4). After filtration, filters were transferred to scintillation vials and scintillation fluid was added. [3H] Spiperone bound radioactivity was determined using a scintillation counter.

### cAMP immunoassay

cAMP content in astrocytes was detected using a cAMP Direct Immunoassay Kit (Colorimetric) (K371-100, BioVision, USA) according to the manufacturer’s protocol. Absorbance of samples was determined by the Multiskan Spectrum (Thermo Scientific) at 450 nm.

### Cell transfection

Astrocytes were allowed to reach 80–90% confluence and transfected with 1 μg of the full-length pcDNA3.1-HA-β-arrestin2 or the pcDNA3.1 empty vector in Opti-MEM (Gibco, USA) using X-tremeGENE HP DNA Transfection Reagent (Roche, Switzerland) for 48 h. Astrocytes were cultured at a confluency of 40–50% and transfected with β-arrestin2 siRNA oligoribonucleotide (sense: GCUUGUGGAGUAGACUUUGTT; antisense: CAAAGUCUACUCCACAAGCTT) (Genepharma, Shanghai, China) using Lipofectamine 3000 reagent (Invitrogen, Life Technologies) in OPTI-MEM-reduced serum medium (Gibco) according to the instructions provided. Six hours later, the transfection mixture was removed and cells were further incubated with normal medium for additional 48 h before stimulation.

### Statistical analysis

All data are expressed as means ± S.E.M. The differences with different treatments and genotypes were determined by one-way or two-way ANOVA, followed by the Tukey’s post hoc test, and the means of two independent samples were compared by *t* test and were considered as statistically significant at *p* < 0.05.

## Results

### Drd2 activation fails to suppress α-Syn-induced neuroinflammation in vivo

**Fig. 1:**
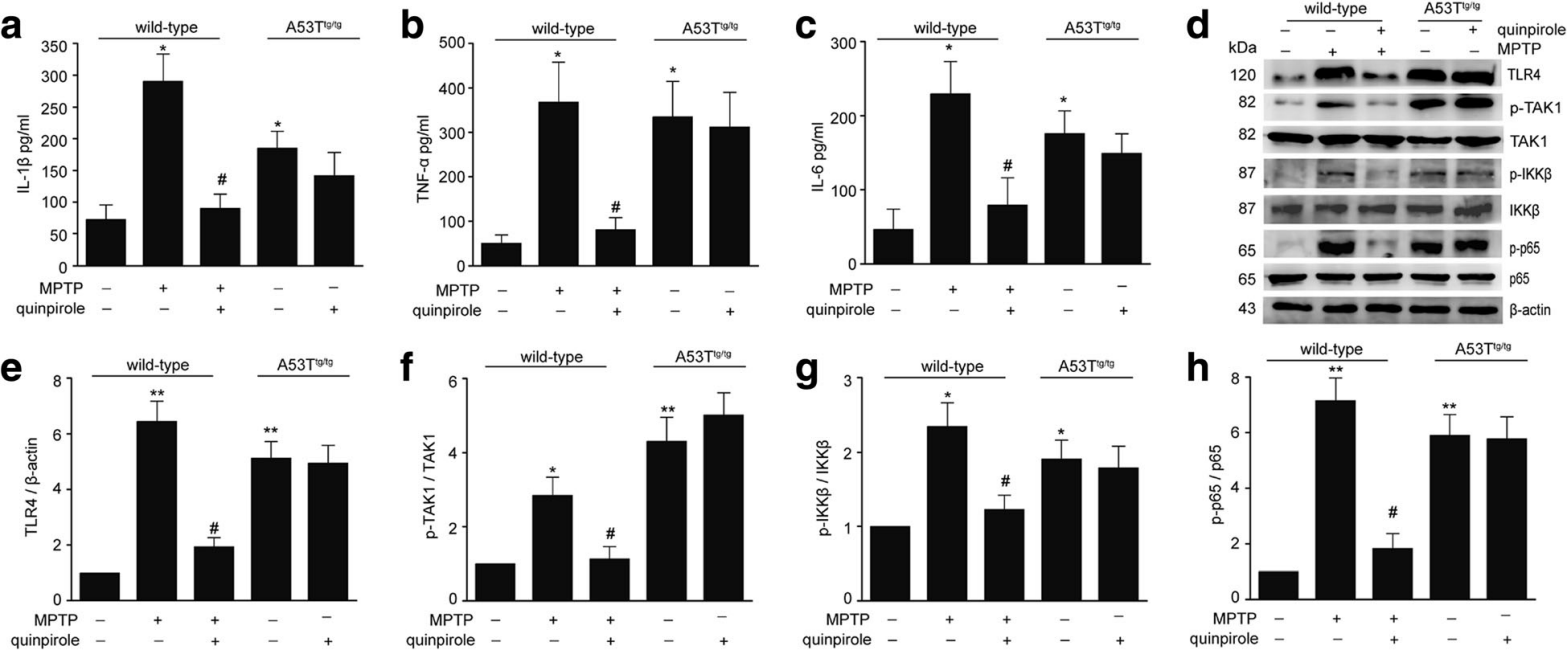
α-Synuclein abolishes the anti-inflammatory role of Drd2 in vivo. **a–c** Quinpirole inhibited the pro-inflammatory mediators production in the SNc of wild-type mice treated with MPTP, but not in A53T transgenic (A53T^tg/tg^) mice. The levels of IL-1β (**a**), TNF-α (**b**), and IL-6 (**c**) evaluated by ELISA in the SNc of wild-type mice and A53T^tg/tg^ mice. **d–h** Quinpirole inhibited the activation of TLR4-NF-κB signal pathways in the SNc of wild-type mice treated with MPTP, but not in A53T^tg/tg^ mice. Representative immunoblot (**d**) and quantitative analysis of TLR4 (**e**), p-TAK1 (**f**), p-IKK (**g**), and p-p65 (**h**) in the SNc of wild-type mice and A53T^tg/tg^ mice. Data are presented as the mean ± S.E.M, *n* = 6, two-way ANOVA, ^*^p < 0.05, ^**^p < 0.01 vs. control group, ^#^*p* < 0.05 vs. MPTP treatment group

To determine the anti-inflammatory role of Drd2 in vivo, A53T^tg/tg^ mice and WT mice were treated with the selective Drd2 agonist quinpirole before MPTP administration. Repeated quinpirole administration resulted in a marked reduction in the levels of pro-inflammatory mediators such as IL-1β, TNF-α, and IL-6 in the SNc of MPTP-treated WT mice. But, quinpirole lost the ability to block the increased production of the pro-inflammatory mediators in A53T^tg/tg^ mice (Fig. 1a–c). Consistently, quinpirole significantly inhibited MPTP-induced the activation of the TLR signaling pathway including the decreased expression of TLR4 and phosphorylation of TAK1 and IKK and reduced nuclear translocation of NF-κB in WT mice, but not in A53T^tg/tg^ mice (Fig. 1d–h). These results indicate that α-Syn abolishes the anti-inflammatory effects of Drd2 in vivo.

### Drd2 agonists fail to inhibit α-Syn-induced neuroinflammation in astrocytes

**Fig. 2:**
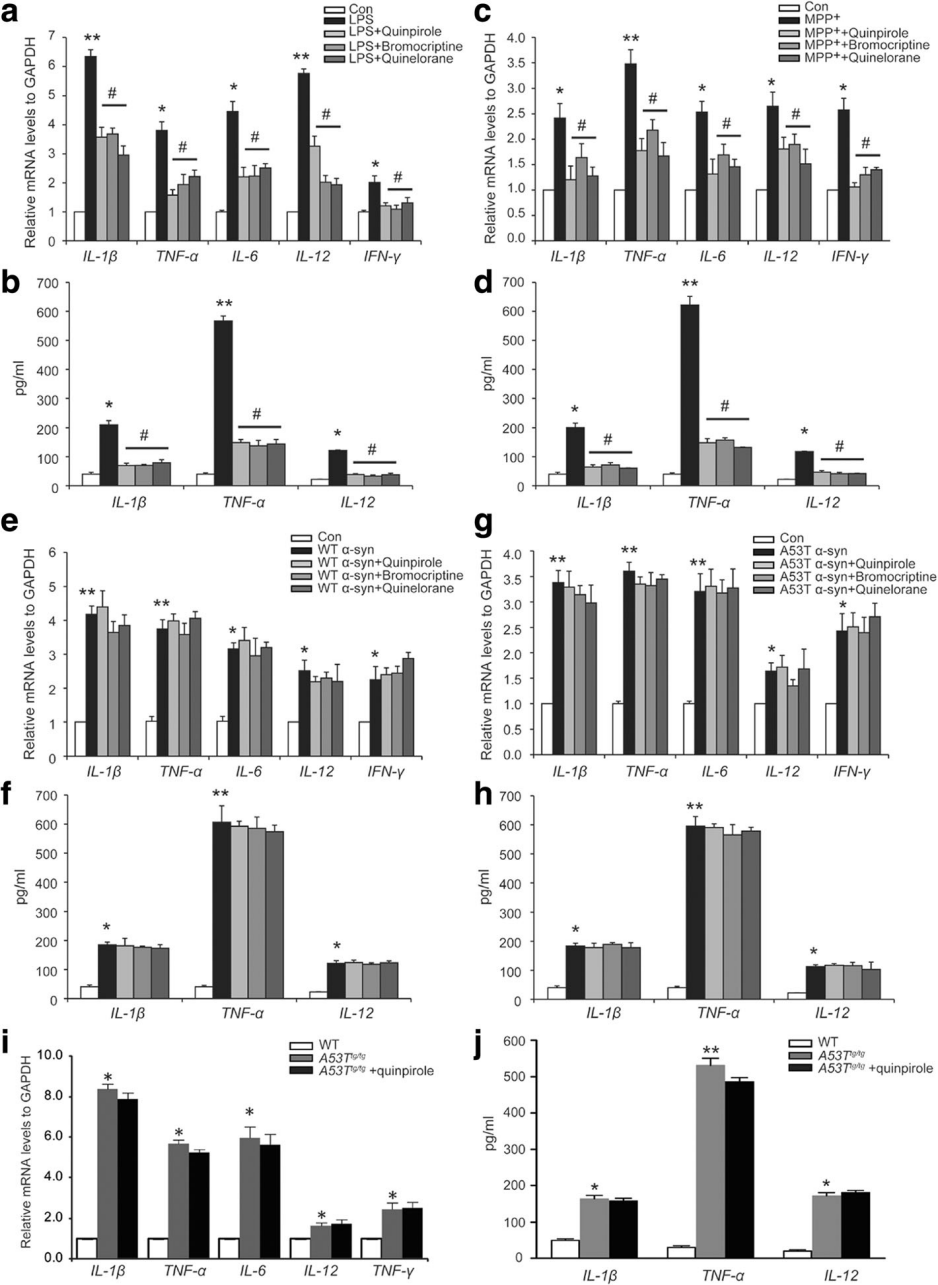
α-Synuclein abolishes the anti-inflammatory role of Drd2 in astrocytes. **a**, **b** Drd2 agonists inhibited LPS-induced the levels of the indicated genes in astrocytes evaluated by qPCR analysis (**a**) and Elisa analysis (**b**). **c**, **d** Drd2 agonists inhibited MMP^+^-induced the levels of the indicated genes in astrocytes evaluated by qPCR analysis (**c**) and Elisa analysis (**d**). **e**, **f** Drd2 agonists failed to suppress the levels of the indicated genes in astrocytes treated with wide-type (WT) α-Syn evaluated by qPCR analysis (**e)** and Elisa analysis (**f**). **g**, **h** Drd2 agonists failed to suppress the levels of the indicated genes in astrocytes treated with A53T mutant α-Syn evaluated by qPCR analysis (**g**) and Elisa analysis (**h**). **i**, **j** Drd2 agonist failed to suppress the levels of the indicated genes in astrocytes from A53T transgenic (A53T^tg/tg^) mice evaluated by qPCR analysis (**i**) and Elisa analysis (**j**). Data are presented as the mean ± S.E.M from four independent experiments, one-way ANOVA, ^*^p < 0.05, ^**^p < 0.01 vs. control group, ^#^*p* < 0.05 vs. LPS/MPP^+^ treatment group

Because our recent study indicates that astrocytes play a critical function in the modulation of neuroinflammation [23], we used primary astrocytes to study the role of Drd2 in neuroinflammation in vitro. Consistent with our data obtained in vivo, Drd2 agonists significantly reduced LPS-induced pro-inflammatory cytokine production including IL-1β, TNF-α, IL-6, IL-12, and IFN-γ (Fig. 2a, b). The anti-inflammatory effect of Drd2 was further studied using MPP^+^, the active metabolite of MPTP, and α-Syn. Treatment with MPP^+^ resulted in marked increase in pro-inflammatory cytokine production in astrocytes, and Drd2 agonists suppressed MPP^+^-induced increase of pro-inflammatory cytokine production (Fig. 2c, d). In marked contrast, either wild-type (WT) α-Syn or A53T mutant α-Syn also increased the production of IL-1β, TNF-α, IL-6, IL-12, and IFN-γ, but quinpirole failed to inhibit the α-Syn-induced elevation of pro-inflammatory cytokines (Fig. 2e–h). Interestingly, other Drd2 agonists including bromocriptine and quinelorane also showed the similar pattern in α-Syn-induced inflammation. Subsequently, the astrocytes from A53T^tg/tg^ mice were used to further confirm the effects of Drd2 in α-Syn-induced neuroinflammation. There were pronounced increases in levels of IL-1β, TNF-α, IL-6, IL-12, and IFN-γ in astrocytes of A53T^tg/tg^ mice compared to WT mice. Consistently, quinpirole could not reduce the levels of pro-inflammatory mediators in astrocytes from A53T^tg/tg^ mice (Fig. 2i, j). These results demonstrate that α-Syn abolishes Drd2-mediated anti-inflammation role in astrocytes.

### α-Syn abolishes the neuroprotective effect of Drd2 in vitro

**Fig. 3:**
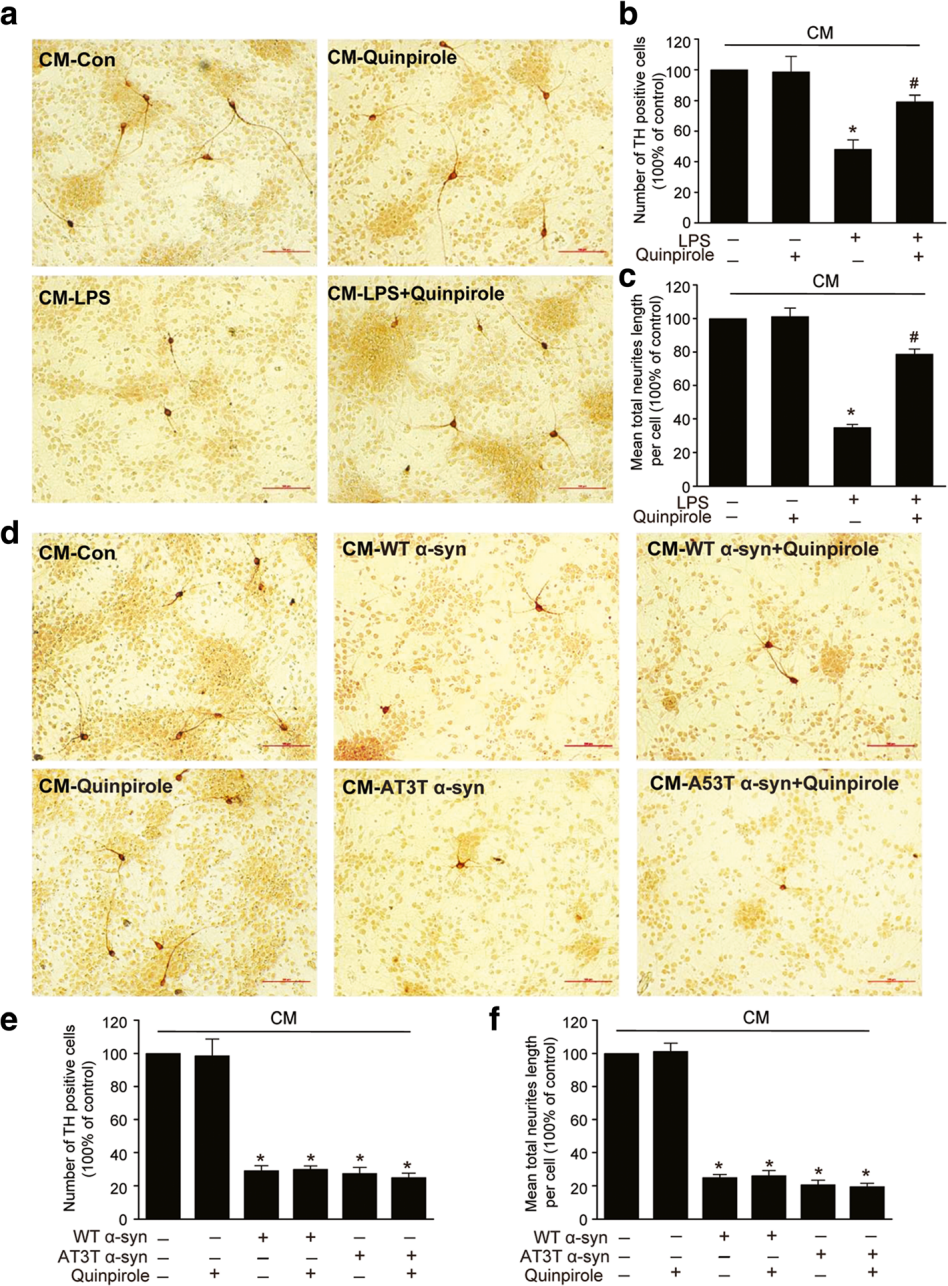
α-Synuclein cancels the neuroprotective effect of Drd2 in vitro. **a–c** Quinpirole protects DA neurons against LPS-induced toxicity. Mesencephalic primary neurons were incubated with the conditioned medium (CM) from astrocyte treated with LPS and quinpirole. The representative pictures of TH^+^ neuron immunostaining (**a**), quantification of TH^+^ cell number (**b**), and mean total neuritis length (**c**). **d–f** Quinpirole failed to protect DA neurons against wide-type (WT) α-Syn or A53T mutant α-Syn-induced toxicity. Mesencephalic primary neurons were incubated with the CM from astrocyte treated with WT α-Syn or A53T α-Syn and quinpirole. The representative pictures of TH^+^ neuron immunostaining (**d**), quantification of TH^+^ cell number (**e**), and mean total neuritis length (**f**). Data are presented as the mean ± S.E.M from four independent experiments, one-way ANOVA, ^*^p < 0.05 vs. control group, and ^#^*p* < 0.05 vs. LPS treatment group

Since neuroinflammation are closely associated with DA neurons death [3], we next studied whether or not Drd2 agonist is neuroprotective against inflammation-induced toxicity of DA neurons. We treated primary culture of SNc TH neurons with the conditioned medium (CM) from astrocyte and quantified the number of TH neurons with immunostaining. Morphological analysis revealed that both the number of TH-positive neurons and the length of neuronal processes were remarkably deceased by CM generated from LPS-treated astrocyte, and this decrease was reversed by treatment with quinpirole (Fig. 3a–c). More importantly, the number of TH-positive neurons and the length of neuronal processes were also inhibited by CM generated from WT α-Syn- or A53T α-Syn-treated astrocyte, but this inhibition was not reversed by quinpirole (Fig. 3d–f). These results suggest that α-Syn abolishes the neuroprotective effect of Drd2 against inflammation-induced DA neurotoxicity.

### α-Syn cancels the suppressive effect of Drd2 on the TLR4-NF-κB pathway in astrocytes

**Fig. 4:**
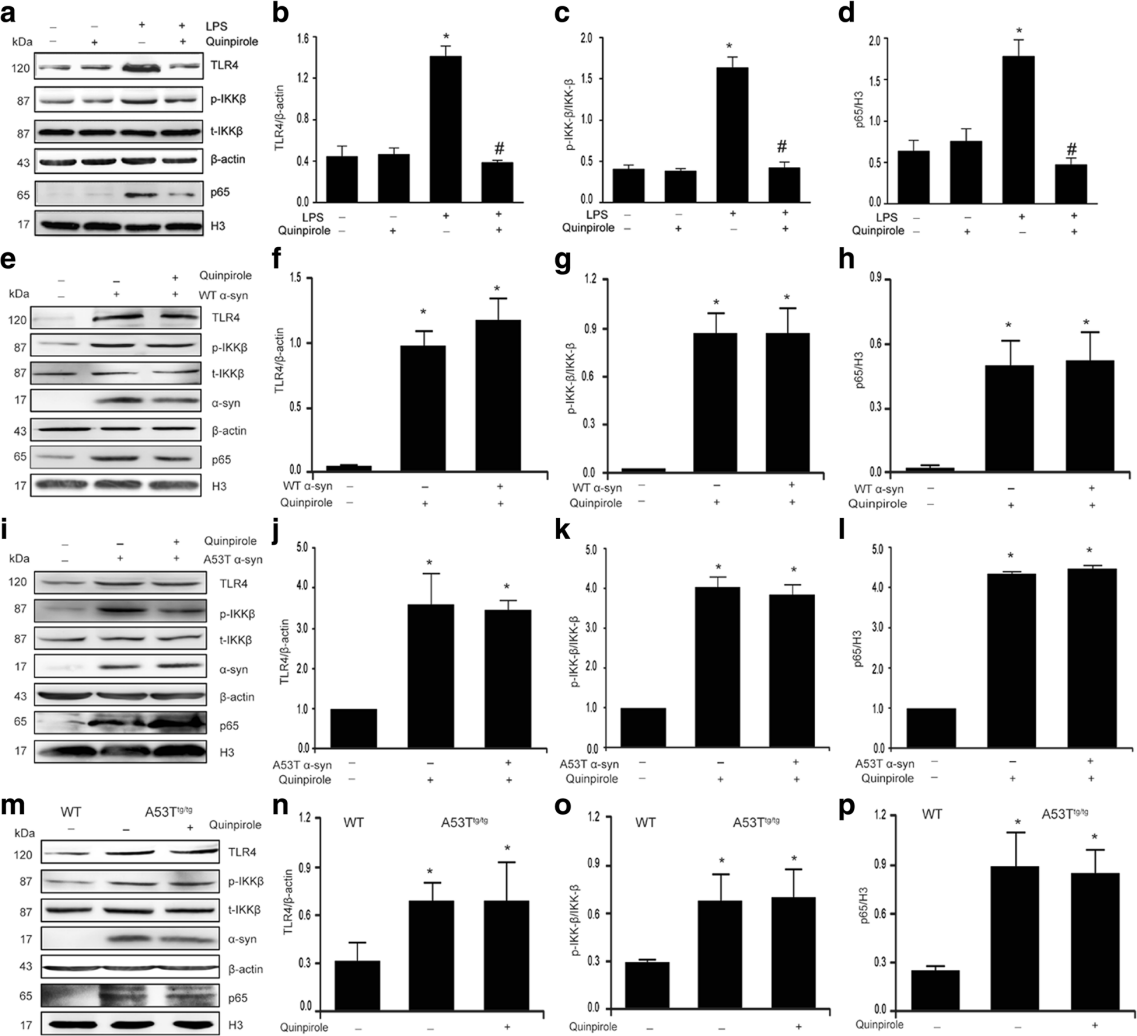
α-Synuclein abolishes Drd2-mediated inhibition of TLR4-NF-κB signaling in astrocytes. **a–d** Quinpirole inhibited the LPS-induced activation of TLR4-NF-κB signaling in astrocytes. Representative immunoblot (**a**) and quantitative analysis of TLR4 (**b**), p-IKK (**c**), and nuclear p65 (**d**) in astrocytes treated with LPS and quinpirole. **e–h** Quinpirole failed to suppress the activation of TLR4-NF-κB signaling induced by wide-type (WT) α-Syn. Representative immunoblot (**e**) and quantitative analysis of TLR4 (**f**), p-IKK (**g**), and nuclear p65 (**h**) in astrocytes treated with WT α-Syn and quinpirole. **i–l** Quinpirole failed to suppress the activation of TLR4-NF-κB signaling induced by A53T mutant α-Syn. Representative immunoblot (**i**) and quantitative analysis of TLR4 (**j**), p-IKK (**k**), and nuclear p65 (**l**) in astrocytes treated with A53T\α-Syn and quinpirole. **m–p** Quinpirole failed to suppress the activation of TLR4-NF-κB signaling in astrocytes of A53T transgenic (A53T^tg/tg^) mice. Representative immunoblot (**m**) and quantitative analysis of TLR4 (**n**), p-IKK (**o**), and nuclear p65 (**p**) in astrocytes of A53T^tg/tg^ mice treated with quinpirole. Data are presented as the mean ± S.E.M from four independent experiments, one-way ANOVA, ^*^p < 0.05 vs. control group, and ^#^*p* < 0.05 vs. LPS treatment group

Because TLR4-NF-κB signaling pathways play a crucial role in inflammation [34], we sought to determine the effect of Drd2 on the activation of TLR4-NF-κB signaling pathways. As shown in Fig. 4a–d, quinpirole suppressed the LPS-induced TLR4 expression, IKK phosphorylation, and NF-κB nuclear translocation. By contrast, both WT α-Syn and A53T α-Syn also markedly increased the expression of TLR4, enhanced the phosphorylation of IKK, and promoted the nuclear translocation of NF-κB, but quinpirole failed to inhibit the α-Syn-induced activation of TLR4-NF-κB signaling pathways (Fig. 4e–l). Identical results were observed in astrocytes from A53T^tg/tg^ mice (Fig. 4m–p). Together, these results suggest that α-Syn cancels the anti-inflammatory effects of quinpirole through interfering with the TLR4-NF-κB signaling pathway activation.

### The anti-inflammation effect of Drd2 is dependent on β-arrestin2-mediated signaling, but not classical G protein pathway

**Fig. 5:**
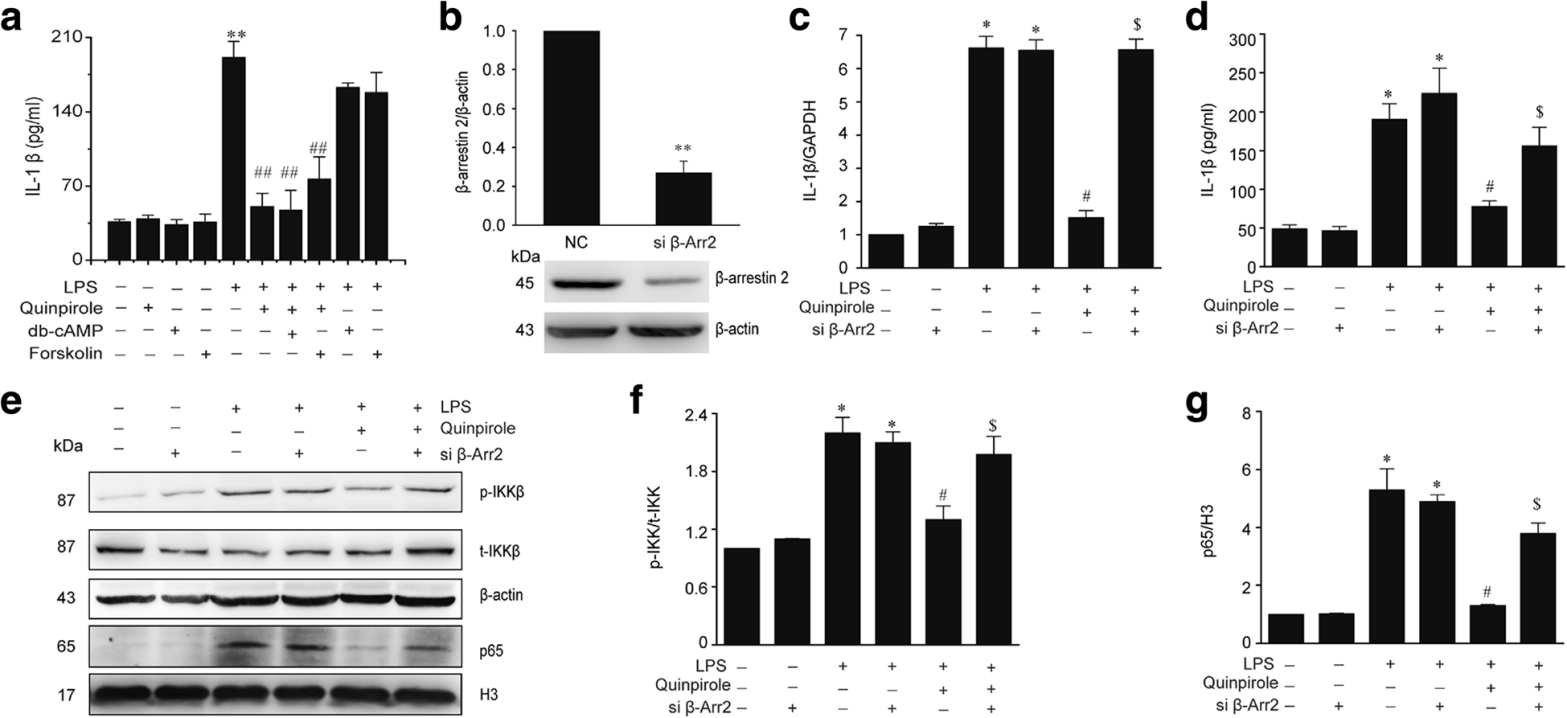
β-Arrestin2 mediates the anti-inflammatory effects of Drd2. **a** Forskolin or dibutyryl cAMP (db-cAMP) failed to abolish the inhibitory effects of quinpirole on the LPS-induced IL-1β production assessed by ELISA in astrocytes. Data are presented as the mean ± S.E.M from four independent experiments, one-way ANOVA, ^**^p < 0.01 vs. control group, and ^##^*p* < 0.01 vs. LPS treatment group. **b–g** Knockdown of β-arrestin2 abolished the anti-inflammatory effects of quinpirole in LPS-treated astrocytes. The astrocytes were transfected with β-arrestin2-specific siRNA (si β-Arr2) for 48 h followed by treated with LPS and quinpirole. b The expression of β-arrestin2 in astrocytes transfected with si β-Arr2 or negative control (NC) for 48 h. Data are presented as the mean ± S.E.M from four independent experiments, *t* test, ^**^p < 0.01 vs. NC group. The levels of IL-1β were determined by qPCR analysis (**c**) and Elisa analysis (**d**). Representative immunoblot (**e**) and quantitative analysis of p-IKK (**f**) and nuclear p65 (**g**) in astrocytes. Data are presented as the mean ± S.E.M from four independent experiments, two-way ANOVA, ^*^p < 0.05 vs. control group, ^#^*p* < 0.05 vs. LPS treatment group, and ^$^*p* < 0.05 vs. LPS + quinpirole group

Activation of Drd2 stimulates classical G protein pathways alongside β-arrestin-dependent signaling [35, 36]. Thus, we explored the possible involvement of both signaling pathways in the observed anti-inflammation effect of Drd2. First, we assessed the role of the classical G protein pathway. As shown in Fig. 5a, AC activator forskolin and dibutyryl cAMP (db-cAMP), a cAMP analogue, could not abolished the anti-inflammation of quinpirole, indicating that Drd2-mediated anti-inflammation is independent of classical G protein pathways. Thus, we speculated that β-arrestin-dependent signaling pathway may mediate the anti-inflammation of Drd2. We then reduced β-arrestin2 expression with β-arrestin2-specific siRNA in astrocyte (Fig. 5b). We found that the anti-inflammatory roles of Drd2 were abolished by knockdown of β-arrestin2 (Fig. 5c, d). In addition, β-arrestin2 knockdown also reversed the inhibitory effects of Drd2 on the activation of TLR4-NF-κB signaling pathway in astrocytes (Fig. 5e–g). These results demonstrate that the anti-inflammatory function of Drd2 in astrocytes depends on β-arrestin2.

### α-Syn reduces the expression of β-arrestin2 in astrocyte

**Fig. 6:**
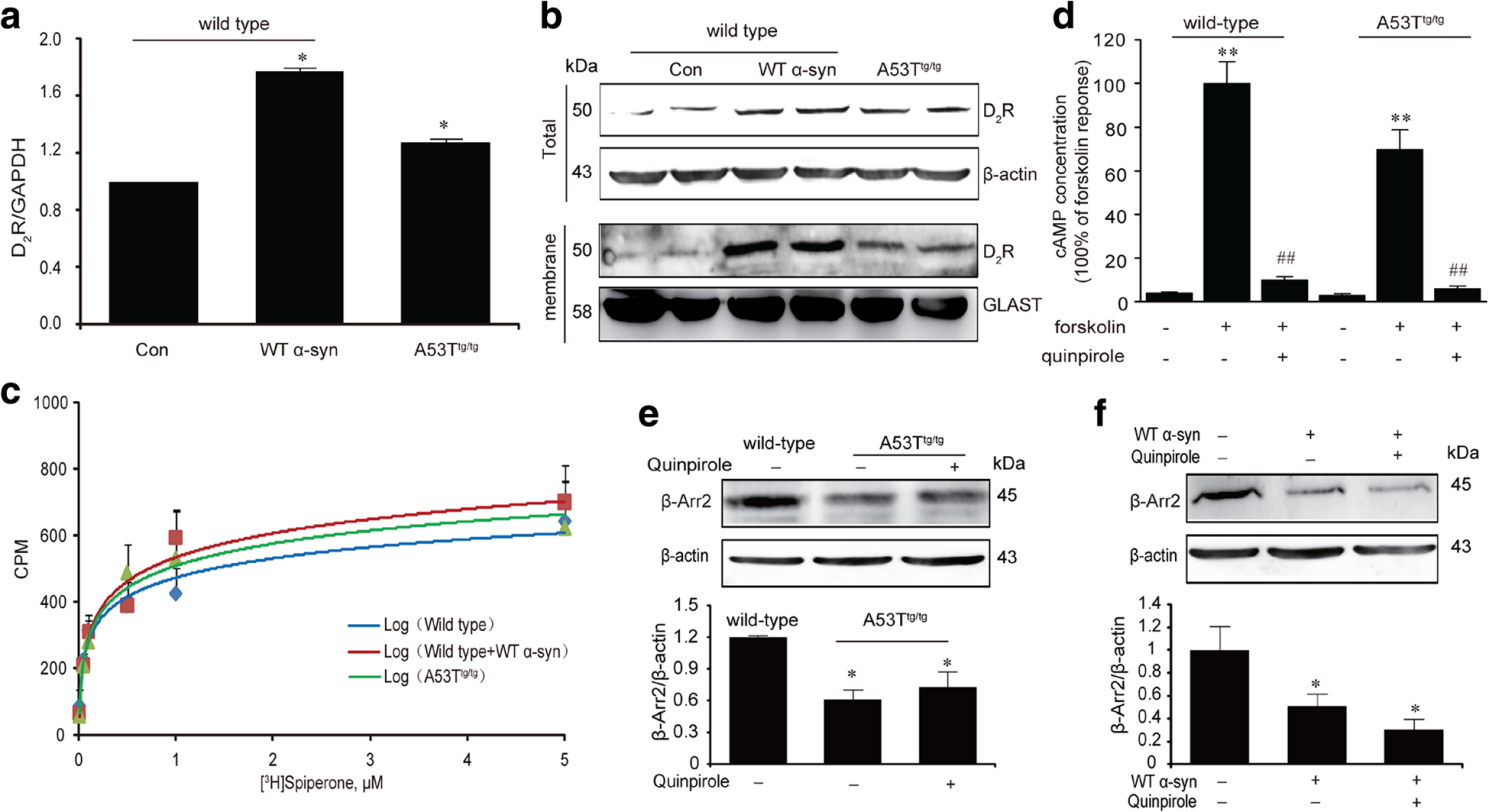
α-Syn reduces the expression of β-arrestin2 in astrocyte. **a–b** α-Syn increased Drd2 expression in astrocytes. The expression of Drd2 in astrocytes treated with wide-type (WT) α-Syn or from A53T transgenic (A53T^tg/tg^) mice were evaluated by the qPCR analysis (**a**) and Western Blot analysis (**b**). Data are presented as the mean ± S.E.M from four independent experiments, *t* test, ^*^p < 0.05 vs. control group. **c** α-Syn failed to influence the binding ability of Drd2 using radioligand binding analysis. **d** α-Syn did not affect the suppressive effects of quinpirole on intracellular cAMP concentration using a cAMP assay kit. Data are presented as the mean ± S.E.M from four independent experiments, two-way ANOVA, ^**^p < 0.01 vs. corresponding control group, ^##^*p* < 0.01 vs. corresponding forskolin treatment group. **e–f** α-Syn reduced the expression of β-arrestin2 in astrocytes. Representative immunoblot and quantitative analysis of β-arrestin2 in astrocytes from A53T transgenic (A53T^tg/tg^) mice (**e**) and treated with wide-type (WT) α-Syn (**f**). Data are presented as the mean ± S.E.M from four independent experiments, two-way ANOVA, ^*^p < 0.05 vs. control group

We first measured the expression and activity of Drd2 in astrocytes from A53T^tg/tg^ and WT mice to determine whether α-Syn cancel the anti-inflammatory effect of Drd2 by reducing membrane Drd2 level or the binding ability of Drd2. As shown in Fig. 6a, b, the membrane Drd2 expression was significantly increased in astrocytes of A53T^tg/tg^ mice, but the binding ability of Drd2 in WT and A53T^tg/tg^ mice showed no difference in the radioligand binding assay (Fig. 6c), indicating that α-Syn did not affect the activation of Drd2 itself. We then probed the downstream signaling pathway of Drd2. In the classical G protein-dependent signaling pathway, Drd2 is associated to Gαi protein to inhibit AC and production of cAMP [37]. Indeed, Fig. 6d showed that active Drd2 suppressed astrocyte cAMP levels in forskolin-stimulated condition, and α-Syn did not influence this process. Thus, we speculated that instead of the classical G protein pathway, it is more likely that α-Syn influence the β-arrestin-dependent signaling pathway. We then detected the expression of β-arrestin2 in astrocytes from WT or A53T^tg/tg^ mice and found that the β-arrestin2 protein expression was significantly decreased in A53T^tg/tg^ astrocytes or WT astrocytes treated with WT α-Syn, and this effect was not reversed by treatment with quinpirole (Fig. 6e–f).

### Increased the β-arrestin2 expression restores the anti-inflammatory effects of Drd2 in α-Syn-induced inflammation

**Fig. 7:**
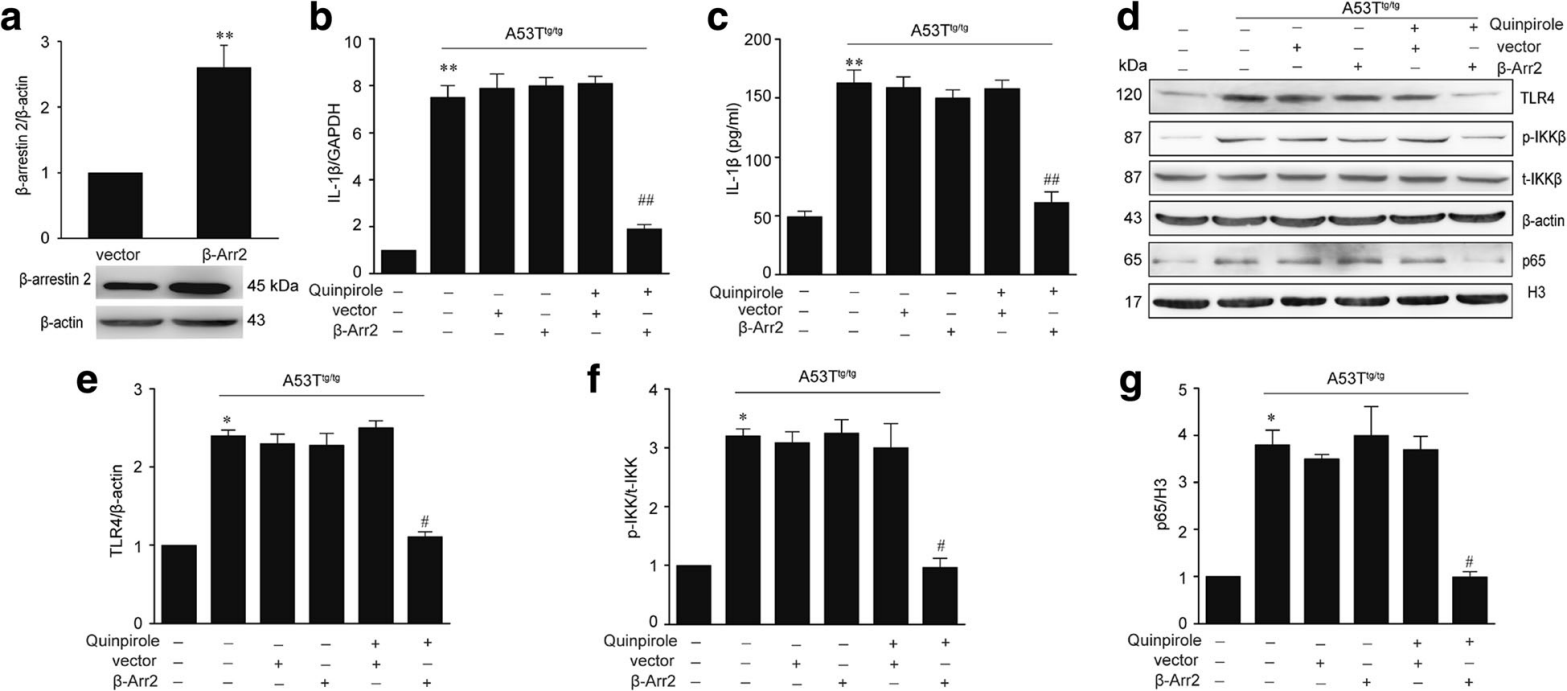
Increased β-arrestin2 expression restores the anti-inflammatory effects of Drd2 in α-Syn-induced inflammation. The astrocytes from A53T transgenic (A53T^tg/tg^) mice were transfected with pcDNA3.1-HA-β-arrestin2 (β-Arr2) or the pcDNA3.1 empty vector (vector) and then treated with quinpirole. **a** The expression of β-arrestin2 in astrocytes transfected with β-Arr2 for 48 h. Data are presented as the mean ± S.E.M from four independent experiments, *t* test, ^**^p < 0.01 vs. vector group. The levels of IL-1β were determined by real-time PCR analysis (**b**) and Elisa analysis (**c**). Representative immunoblot (**d**) and quantitative analysis of TLR4 (**e**), p-IKK (**f**), and nuclear p65 (**g**) in astrocytes. Data are presented as the mean ± S.E.M from four independent experiments, two-way ANOVA, ^*^p < 0.05, ^**^p < 0.01 vs. control group, ^#^*p* < 0.05, ^##^*p* < 0.01 vs. quinpirole + vector group

Our preceding data have demonstrated that α-Syn reduces the expression of β-arrestin2 in astrocytes. We then determined if enhanced expression of β-arrestin2 could restore the anti-inflammation role of Drd2 in α-Syn-induced inflammation. As shown in Fig. 7a–c, β-arrestin2 overexpression indeed restored quinpirole’s suppressive effect on IL-1β production in the A53T^tg/tg^ mice astrocyte. As expected, β-arrestin2 overexpression also rescued quinpirole’s ability to inhibit the activation of the TLR4-NF-κB signaling pathway, including downregulated expression of TLR4, decreased phosphorylation of IKK, and reduced nuclear expression of NF-κB p65 in astrocyte of A53T^tg/tg^ mice (Fig. 7d–g). These results indicate that α-Syn cancels the anti-inflammatory effect of quinpirole through downregulation of β-arrestin2.

### α-Syn disrupts the β-arrestin2-TAB1 interaction in astrocytes

**Fig. 8:**
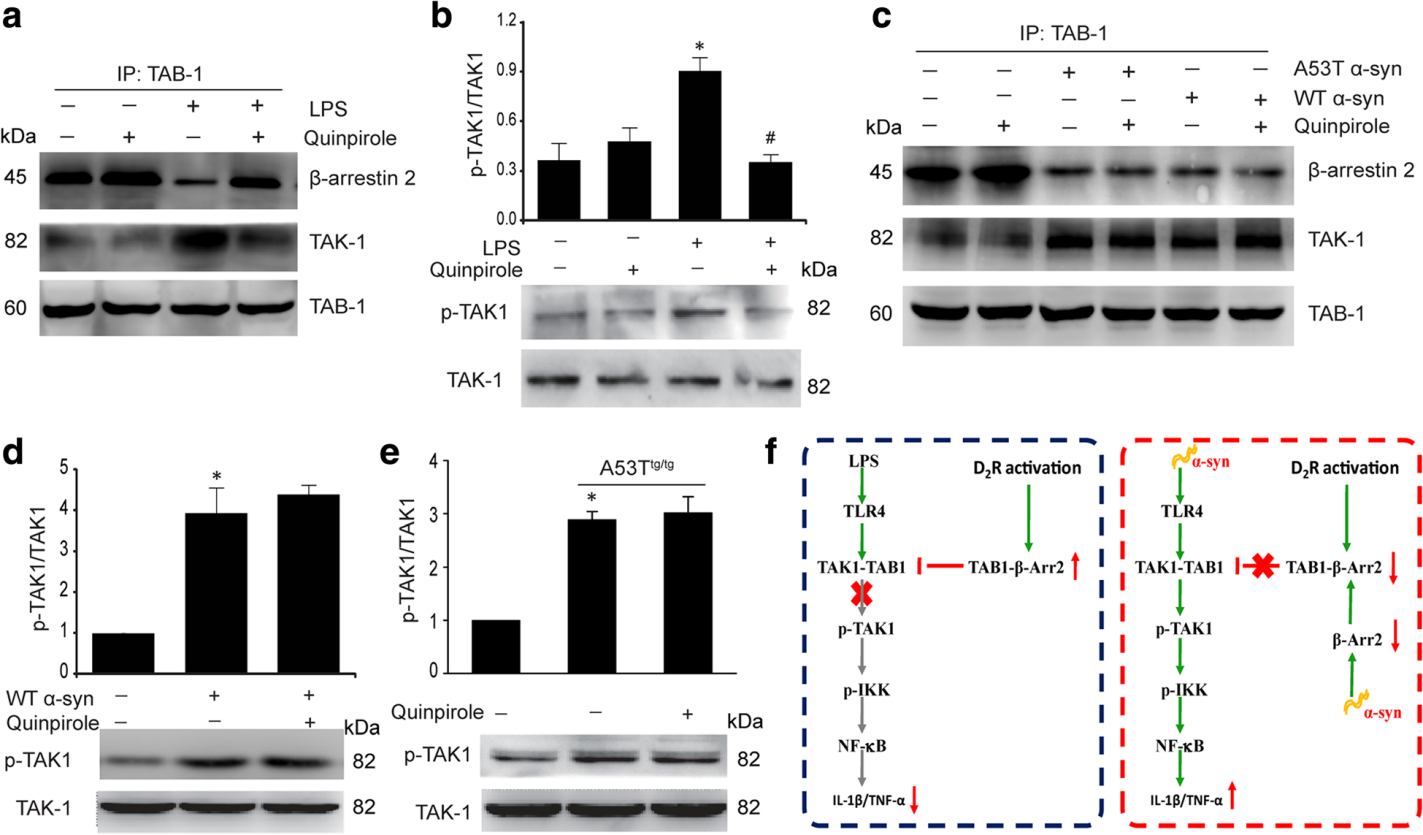
α-Synuclein disrupts the β-arrestin2-TAB1 interaction in astrocytes. **a** Quinpirole enhanced the TAB1-β-arrestin2 interaction and inhibited the TAB1-TAK1 interaction in astrocytes. The interaction of TAB1 with β-arrestin2 and TAK1 in astrocytes treated with quinpirole for 1 h prior to addition of LPS measured by co-IP. **b** Quinpirole inhibited the LPS-induced TAK1 activation (p-TAK1) evaluated by Western blot analysis. Data are presented as the mean ± S.E.M from four independent experiments, one-way ANOVA, ^*^p < 0.05 vs. control group, and ^#^*p* < 0.05 vs. LPS treatment group. **c** α-Syn reduced the TAB1-β-arrestin2 interaction and enhanced the TAB1-TAK1 interaction in astrocytes. The interaction of TAB1 with β-arrestin2 and TAK1 in astrocytes treated with quinpirole for 1 h prior to addition of wide-type (WT) α-Syn or A53T mutant α-Syn measured by co-IP. **d**–**e** Quinpirole failed to inhibit the α-Syn-induced TAK1 activation (p-TAK1) evaluated by Western blot analysis. Representative immunoblot and quantitative analysis of p-TAK1 in astrocytes treated with WT α-Syn (**d**) or in A53T transgenic (A53T^tg/tg^) mice astrocytes (**e**). Data are presented as the mean ± S.E.M from four independent experiments, two-way ANOVA, ^*^p < 0.05 vs. control group. **f** A model depicting the roles of α-Syn in disrupting the D2R/β-arrestin2 anti-inflammatory pathway via disassembling of TAB1-β-arrestin2 complex

The association of TAK1 with TAB1 is a prerequisite for the activation of TAK1 and TLR4 pathway [38]. To dissect the molecular mechanisms underlying anti-inflammation of Drd2, we first measured the interaction between the three proteins TAK1, TAB1, and β-arrestin2 in co-IP assays. As shown in Fig. 8, LPS markedly inhibited TAB1-β-arrestin2 interaction, increased the TAK1-TAB1 association, and enhanced the phosphorylation of TAK1 (p-TAK1), and these effects were reversed by quinpirole treatment (Fig. 8a–b). In contrast, α-Syn also reduced the TAB1-β-arrestin2 interaction, augmented the association of TAB1 with TAK1, and promoted p-TAK1, but quinpirole could not reverse these effects (Fig. 8c–e). These findings demonstrate that α-Syn abolishes the anti-inflammatory effect of Drd2 by disrupting TAB1’s anti-inflammatory association with β-arrestin2 and enhancing TAB1’s pro-inflammatory association with TAK1.

## Discussion

The most important finding presented here is that α-Syn abolishes anti-inflammatory effects of Drd2 in vivo and in vitro. Using well-established LPS-mediated inflammatory model, we demonstrated that Drd2 activation is protective against inflammation-induced degeneration of DA neurons and that this protection is mediated through inhibition of neuroinflammation in astrocytes. However, this protective function of Drd2 on DA neurons in response to α-Syn-induced inflammation was abolished. Mechanistic studies show that anti-inflammation of Drd2 is mediated by the inhibition of the TLR4-TAK1-NF-κB axis in astrocytes, and this function is independent of the conventional GPCR/cAMP signaling pathways; rather, this inhibition is dependent on β-arrestin2, which shows a novel mode of action for the Drd2 in regulating CNS inflammatory conditions. Moreover, α-Syn abolishes anti-inflammation role of Drd2 via downregulation of β-arrestin2 expression and disrupting the interaction of β-arrestin2 and TAB1.

In neurons, the dopamine /Drd2 system’s effect on locomotion and behavioral changes has been well studied [39]. Abnormal dopamine signaling plays a role in various neuropathies such as schizophrenia, depression, and PD [40–42]. However, how Drd2 functions in glial cells is still poorly understood. Our early study showed that astrocytic Drd2 deficiency increased inflammatory response through downregulation of αB-crystallin [23]. Here, we found that Drd2 agonist quinpirole inhibited the LPS/MPP^+^-induced increase of inflammatory mediators IL-1β, TNF-α, IL-6, IL-12, and IFN-γ, whereas quinpirole lost this ability in α-Syn-treated atrocytes. We further showed that quinpirole reduced the number of LPS-induced neuronal deaths. However, this protective function of quinpirole on neurons in response to α-Syn-induced inflammation was abolished, demonstrating that the α-Syn abolishes the neuroprotective effect of Drd2 against inflammation-induced DA neurotoxicity. Equally potent efficacy of quinpirole’s action in inflammation was also observed in MPTP PD model. We also found that quinpirole suppressed the inflammatory cytokine production in WT mice of MPTP PD model, but lost the ability to block the increased production of the pro-inflammatory genes in A53T^tg/tg^ mice. In summary, both our in vivo and in vitro results clearly demonstrate that α-Syn abolish the anti-inflammatory role of Drd2. On the basis of these findings, we speculate that diffuse α-Syn in the CNS may be the saboteur that impedes the therapeutic effect of dopamine receptor in late PD.

Increasing evidence from both in vivo and in vitro studies suggests that Drd2 possesses important immunomodulatory potential [24, 43, 44]. Our studies provide a novel pathway that is different from the conventional Drd2/cAMP-dependent pathway mediated by Drd2 activation. These findings also provide opportunities for novel therapeutic approaches in inflammation-mediated CNS disorders such as PD. In the present study, it is clear that Drd2-mediated anti-inflammatory effects are not cAMP dependent because AC activators or cAMP analog could not block the anti-inflammatory activity of Drd2. For this reason, we looked for an alternative pathway mediating Drd2-related protective effects. Arrestins, originally discovered as terminators of GPCR signaling by facilitating desensitization and internalization of GPCR, recently have been recognized as multifunctional adaptor/scaffold proteins in regulating cellular processes such as chemotaxis, apoptosis, metastasis, and inflammation [45]. β-arrestin2 has been found to be a negative regulator of inflammatory responses in monocytes, macrophages, and microglia [28, 46, 47], but its role in astrocyte remains unknown. Our studies demonstrate that Drd2-mediated anti-inflammatory effects are dependent on β-arrestin2 in astrocytes. Drd2 activation enhances β-arrestin2’s association with TAB1. This competitively blocks TAB1’s interaction with TAK1 and TAK1’s subsequent phosphorylation, which attenuates the TLR4-dependent signaling pathway (Fig. 8f). This suggests that a novel GPCR-coupled signaling pathway may be involved and suggests that β-arrestin2 may represent a new target for anti-inflammatory therapy in NDD including PD.

Given the importance of Drd2-mediated anti-inflammatory effects in treatment of PD, it is urgent to mechanistically explore how α-Syn abolishes the anti-inflammation of Drd2 to develop such therapeutic interventions. Since Drd2 agonist exerts its biological functions by activation of Drd2 through G protein-dependent cellular processes or alternative G protein-independent signaling pathways [36], we first suppose that α-Syn may reduce membrane Drd2 level or the binding ability of Drd2. Surprisingly, we found that α-Syn actually increased membrane Drd2 level and could not inhibit the binding ability of Drd2, suggesting that α-Syn did not affect the activation of Drd2 itself. We then probed the downstream signaling pathway of Drd2. In the classical G protein-dependent signaling pathway, Drd2 is associated to Gi/o protein to inhibit the production of cAMP [37]. We showed that α-Syn failed to influence cellular cAMP level. For this reason, we seek an alternative G protein-independent pathway. We found that α-Syn decreased the expression of β-arrestin2 and suppressed β-arrestin2’s association with TAB1. This enhances TAB1’s interaction with TAK1 and TAK1’s subsequent phosphorylation, which promotes the TLR4-dependent signaling pathway and abolishes Drd2 inhibition of pro-inflammatory cytokine production (Fig. 8f). This was further supported by our finding that overexpression of β-arrestin2 could restore the anti-inflammation role of Drd2 in α-Syn-induced inflammation. These results demonstrate that α-Syn abolishes the anti-inflammatory effects of Drd2 via β-arrestin2. Over all, Drd2 activation increased the β-arrestin2-TAB1 interaction and this competitively blocks TAB1’s association with TAK1 and subsequent phosphorylation of TAK1, which attenuates the TLR4-dependent signaling pathway and inhibits the production of inflammatory mediators. α-Syn abolishes the anti-inflammatory effects of Drd2 via downregulation expression of β-arrestin2 and disrupting the association of β-arrestin2 with TAB1 (Fig. 8f).

## Conclusions

The present study unravels a novel mechanism of Drd2-mediated suppression of inflammatory response in a β-arrestin2-dependent pathway, and α-Syn abolishes the anti-inflammatory effects of Drd2 via β-arrestin2 in astrocytes. In addition, our study extends our understanding on β-arrestin2-biased Drd2 signaling and provides potential new therapeutic avenues for PD.

## Abbreviations

A53T^tg/tg^: A53T transgenic
CNS: Central nervous system
DA: Dopaminergic
ELISA: Enzyme-linked immunosorbent assay
IL-1β: Interleukin-1 beta
IL-6: Interleukin-6
MPTP: 1-Methyl-4-phenyl-1, 2, 3, 6-tetrahydropyridine
NDDs: Neurodegenerative diseases
NF-κB: Nuclear transcription factor kappa B
PD: Parkinson’s disease
RT-qPCR: Real-time quantitative polymerase chain reaction
SNc: Substantia nigra compacta
TAB1: Transforming growth factor-beta-activated kinase 1 (TAK1)-binding protein 1
TAK1: Transforming growth factor-beta-activated kinase 1
TH: Tyrosine hydroxylase
TNF-α: Tumor necrosis factor alpha
WT: Wild-type LPS Lipopolysaccharide
α-Syn: α-Synuclein
